# GENETIC PREDISPOSITION TO ACCELERATED BIOLOGICAL AGES PREDICTED BY BIOCHEMICAL MARKERS

**DOI:** 10.1093/geroni/igz038.3442

**Published:** 2019-11-08

**Authors:** Chia-Ling Kuo, Luke C Pilling, Zuyun Liu, Morgan E Levine

**Affiliations:** 1 University of Connecticut, Farmington, Connecticut, United States; 2 University of Exeter, Exeter, United Kingdom; 3 Department of Pathology, Yale School of Medicine, New Haven, Connecticut, United States; 4 Yale University School of Medicine, New Haven, Connecticut, United States

## Abstract

Biological ages predicted by biochemical markers (biomarkers) outperform other measures in predicting a variety of aging outcomes. Several have been developed in recent studies, and there is evidence that each may independently predict mortality. While the included biomarkers are disease-associated, it is unclear what aspects of aging are captured. We aimed to understand and quantify genetic predisposition to accelerated biological ages, determined based on two measures, PhenoAge (9 biomarkers plus chronological age, Levine et al. 2018) and BioAge (7 biomarkers plus chronological age, Levine 2013). We performed genome-wide scans using the UK Biobank data (n=107,460 for PhenoAge, n=98,446 for BioAge). The SNP-based (single nucleotide polymorphism) heritability estimates were 14.45% and 12.39% for PhenoAge and BioAge, respectively. Both shared the strongest signal in the APOE region, with opposite associations with e2 and e4 alleles. e2 was associated with younger BioAge but older PhenoAge. e4 was associated with older BioAge but younger PhenoAge. BioAge was highly genetically correlated with its element of systolic blood pressure (rg=0.84) and the genetic correlation between PhenoAge and red blood cell distribution width was 0.65. Previous genome-wide association study findings of the top hits suggest that BioAge mostly captures cardiac aging but PhenoAge has more to do with inflammatory aging. The results are consistent with SNP clusters by associations with a broad range of aging traits, including an independent cluster with SNPs near the APOE. Genetic risk scores will be created to quantify the genetic predisposition and will be tested for associations with numerous aging traits.

